# Microgreens as a Component of Space Life Support Systems: A Cornucopia of Functional Food

**DOI:** 10.3389/fpls.2017.01587

**Published:** 2017-09-12

**Authors:** Marios C. Kyriacou, Stefania De Pascale, Angelos Kyratzis, Youssef Rouphael

**Affiliations:** ^1^Department of Vegetable Crops, Agricultural Research Institute Nicosia, Cyprus; ^2^Department of Agricultural Sciences, University of Naples Federico II Portici, Italy

**Keywords:** carotenoids, functional salad, light-emitting diodes (LED), microgravity, space farm

## Prolonged presence in space and the need for a functional diet

The future of space missions and extended human presence in space requires the ability to provide proper dietary intake for space travelers with minimal resupply from the Earth, as food and food packaging currently represent a significant burden on space mission consumables (Perchonok et al., 2012). This is critical for sustaining an optimal nutritional status for space travelers and for mitigating stress effects from long-duration space travel, including weight loss, hematological changes, and space radiation-induced oxidative cytotoxic stress, protein oxidation, increased muscle proteolysis, impairment of eye health and changes in the central nervous system (Kennedy et al., 2007; Vergari et al., 2010; Cohu et al., 2014). Such effects are further linked to emotional volatility, psychological stress, and depression among the crew (Rabin et al., 2005). Prevention of deleterious phenomena that accelerate tissue lethality must include targeted intake of whole food-based antioxidants rather than supplements (Wan et al., 2006). These comprise fresh plant sources produced aboard during mission, thereby providing emotional along with nutritional support to space travelers.

For instance, the consumption of carotenoids through whole-food-based diet is a recommendable protective measure since the human body is unable to produce any of the major photoprotective carotenoids considered essential for human vision: β-carotene as precursor of retinal constituent vitamin A, and zeaxanthin and lutein for protecting the eyes by absorbing excess light intensity (Cohu et al., 2014). Production of bioactive and particularly carotenoid-rich vegetables as part of Space Life Support Systems (SSLSs) remains a critical goal for future space missions (Perchonok et al., 2012). Awareness of the importance of fresh functional food in physically and mentally fortifying crews during missions has been growing among space mission participants (Vergari et al., 2010). It is therefore critical to incorporate in SLSSs plant-based fresh functional food production to support human presence during long-distance space travel or extra-planetary habitation.

## Space farm essentials and constraints

Plant growth under space conditions is faced with important constraints unknown to Earth-based farming systems and beyond our current understanding of plant physiological responses in terrestrial environments. These include, among others, exposure to high levels of cosmic radiation, lack of a unilateral gravity vector, growth adapted to limited chamber space, reduced nutrient sustainability, and lack of convection (Vandenrbrink and Kiss, 2016). Space farming will be conducted in controlled environment chambers that must infringe minimally on power, volume, and mass resources shared with crews and used for maintaining spacecraft in stable orbit (Poulet et al., 2016). In bioregenerative closed systems, plants are destined to produce food, regenerate the air by removing CO_2_ and producing O_2_, and recycle water through transpiration. A major challenge for adapting Earth-based agricultural practices aboard spacecraft or in planetary bases is reduced gravity (or microgravity) that impacts fluid and gas distribution around the plants (Kuang et al., 2000). Reduced mass transport and absence of buoyancy-dependent convective transport are responsible for thick boundary layers forming around plant organs, allowing the build-up of volatile compounds deleterious for plant growth, such as ethylene, and reducing oxygen bioavailability (Monje et al., 2003). Power-assisted forced convection is required as reduced gravity-mediated oxygen transport may result in hypoxic conditions around plant organs whenever the rate of respiration exceeds the rate of oxygen mass transfer to the respiring organs; moreover, transpiration rates tend to increase under hypobaric conditions. Low irradiance levels (≤300 μmol m^−2^ s^−1^) is also among the serious constraints imposed on space farming as supplemental lighting is considered a highly energy demanding subsystem of the space farm (Salisbury and Bugbee, 1988).

Gravity is not an absolute requirement for any step in the plant life cycle as complete ontogenesis from seed to seed has been demonstrated in microgravity conditions (Kuang et al., 2000). Tomato seeds formed under simulated microgravity were biologically and functionally complete (Colla et al., 2007). However, seeds formed in space demonstrated retarded deposition of reserves during development (Musgrave et al., 2005), while reduced storage reserve mobilization and hypoxia-induced changes in mitochondrial size and shape, and in starch grain size and distribution was reported during *Brassica* early seedling growth along (Musgrave, 2002). Seeds of *Brassica rapa* L. produced in space were smaller, had lower dry weight, were deficient in protein and presented differences in bioactive phytochemicals, such as glucosinolates, compared to ground control seeds (Musgrave et al., 2005). Moreover, morphological and growth characteristics of dwarf tomato plants were modified during microgravity simulation treatment, presenting spreading growth, increased internode length, reduced fruit yield, fruit size, leaf area, leaf dry weight, fruit dry weight, total dry weight, foliar amounts of chlorophylls and carotenoids as well as reduced fruit sugar and dry mater contents (Colla et al., 2007).

## Ideal candidates for the astronaut's functional salad

The choice of crops may partly hold the answer to several of the challenges facing seed-to-seed production in microgravity. Crop criteria established for plants grown in space include: the ratio of edible mass to total biomass (harvest index), crop efficiency (per unit area, time, and volume), potential yield (edible mass and O_2_ and H_2_O production), and the crop's horticultural requirements (planting, harvesting, pollination, processing needs; Berkovich et al., 2004; Yamashita et al., 2009). Salad crops present the highest harvest indices (≈90%) among candidate crops, and low water uptake/transpiration ratio which translates into high humidity input into the space flight environment that can be harnessed, but they cannot be part of a closed system using recycled gray water (Anderson et al., 2015); moreover, they are characterized by low O_2_ production and CO_2_ consumption rates, i.e., low biomass fixation. Salad crops are highly suitable for chamber cultivation, they are easy to cultivate, they have short growth cycles, they are low ethylene producers and can be picked and eaten fresh, requiring minimal horticultural input from the crew (Chunxiao and Hong, 2008). Moreover, growing salad crops is easily adaptable to the needs of a diverse and renewed diet while adding a palatable and bioactive aspect to it.

A new class of speciality salad crops valued for their color and flavor enhancing properties but also for their rich phytonutrient content are microgreens (Kyriacou et al., 2016; Bulgari et al., 2017). Produced from the seeds of vegetables, herbs, or grains, including wild or even ornamental species, microgreens have a brief, species-dependent production cycle, of 1–3 weeks from seed germination (Xiao et al., 2012). They are harvested at soil level, when cotyledons are fully expanded and the first pair of true leaves has emerged (Sun et al., 2013). They have recently gained immense popularity as culinary ingredients of novel gastronomic trends (Koppertcress, 2016). Candidate genotypes are expanding based on sensory and health criteria, however, currently exploited are mostly species from the *Brassicaceae, Asteraceae, Chenopodiaceae, Lamiaceae, Apiaceae, Amarillydaceae, Amaranthceae*, and *Cucurbitaceae* families (Ebert, 2012). Compared to their mature-leaf counterparts, microgreens contain higher amounts of important phytonutrients (ascorbic acid, β-carotene, α-tocopherol, and phylloquinone) and minerals (Ca, Mg, Fe, Mn, Zn, Se, and Mo) and lower nitrates (Xiao et al., 2012; Pinto et al., 2015). Seeds are demanded in large quantity and high quality, thus constitute a major cost of microgreens production (Di Gioia et al., 2015). Although, foodborne outbreaks have not been associated with the consumption of microgreens, seeds should receive precautionary sanitary treatments for eliminating pathogenic bacteria (Xiao et al., 2014, 2015). Ease of germination varies among microgreens species, with slow germinating species benefiting from brief pre-sowing treatments that help standardize and shorten their production cycle (Lee et al., 2004). Sowing rate depends on average seed weight, estimated germinability and targeted crop density, ranging from 1 seed/cm^2^ for large-seeded species (e.g., pea, chickpea, sunflower), up to 4 seeds/cm^2^ for small-seeded species (e.g., arugula, watercress, mustard; Di Gioia and Santamaria, 2015). Microgreens are ideal for space flight environments as they can be harvested directly by crew members, ensuring freshness and high quality. Their production can be implemented on static, shallow substrates with little or no nutrient supplementation, and this alleviates problems of poor crop performance associated with low O_2_ and nutrient solubility in microgravity hydroponic systems (Perchonok et al., 2012). Synthetic fibrous media can be used and fortified to improve the nutritional value of microgreens (Nyenhuis and Drelich, 2015), furthermore they eliminate the need to prepare and administer complete nutrient solutions and allows transpired water to be recycled to the root module. Bioactive content is usually pronounced in less palatable microgreens species, such as red cabbage (*Brassica oleracea* L. var. *capitata*), sorrel (*Rumex acetosa* L.), peppercress (*Lepidium bonariense* L.), but also in some species of more agreeable taste such as cilantro (*Coriandrum sativum* L.) and amaranth (*Amaranthus hypochondriacus* L.; Xiao et al., 2012).

A major constraint of food production in SLSSs is the high demand for power which antagonizes other space shuttle subsystems (Perchonok et al., 2012). Providing efficient and optimal, in terms of intensity and spectral quality, lighting for crops in spaceflight environments has been increasingly feasible through the introduction of light-emitting diode (LED) technology (Wheeler, 2004; Bourget, 2008). Using LEDs instead of metal halide, fluorescent, incandescent, and high-pressure sodium lamps can reduce power demand per unit of growing area by up to one order of magnitude (Poulet et al., 2014, 2016). Microgreens have a lower demand for photon flux compared to long-cycle crops, thus are ideally adapted to chamber environments. Moreover, modulating the photon flux, photoperiod and especially the wavelength can be an effective means of achieving compound-specific improvements in the functional quality of microgreens and decrease in the levels of anti-nutrients (Kyriacou et al., 2016). For example, supplemental green light improved carotenoid content (β-carotene and lutein/zeaxanthin ratio) in mustard microgreens, while standard blue/red/far red LED illumination increased the levels of carotenoids in red pak and tatsoi (Brazaityté et al., 2015). Even brief (3 d) preharvest application of supplementary red LED was efficacious in improving the antioxidant profiles of several microgreens species (Samuolienė et al., 2012). Red and blue lights, or their mixture, were found more effective than white and yellow in reducing undesirable nitrates in several species (Ohashi-Kaneko et al., 2007; Qi et al., 2007). However, the exact mechanisms behind spectral quality-induced changes on bioactive compounds are far from elucidated and deserve further attention.

## Challenges ahead

In the near future, space exploration will inevitably expand and its food supply system must also continue to evolve. In this perspective, microgreens could be considered a resilient phytochemical factory for the dietary and psychological needs of crew members in orbital flights and platforms. However, as components of bioregenerative life support systems, plants must provide food to the astronauts as well as sufficient photosynthetic CO_2_ fixation, O_2_ regeneration and transpirational filtering, and recycling of water. Despite their high harvest index, microgreens are characterized by low biomass fixation and consequently low O_2_ generation. To a certain extent, this can be maximized by growing microgreens in multi-tiered systems, thereby also increasing space efficiency and water transpiration. Moreover, the need for higher O_2_ generation can be met by growing microgreens along with larger edible crops characterized by higher biomass fixation but, usually, lower harvest index. Microgreens also provide an easy platform for widening space crop genetic diversity since the diverse cultural needs of long-cycle crops are avoided. Such broad genetic basis is desirable both from a nutritional and sensorial standpoint but also as a means of fending off SLS systems from potential plant disease outbreaks. Although, growing microgreens on artificial media is usually problem-free, food safety, and healthy crop stand necessitate appropriate precautionary measures such as using only high quality certified seed produced and handled under conditions that minimize potential contamination with pathogenic organisms, sanitary maintenance of facilities employed for growing microgreens, pre-sowing treatment of seed with appropriate sanitizers such as calcium hypochlorite, testing, and effective disinfection of irrigation water. Finally, an important factor that warrants further research is the effect of space radiation on seed germinability. Although, this is a horizontal factor influencing both seed-to-seed and short-cycle crops, ensuring seed viability and germinability is even more critical for high seed rate crops such as microgreens.

## Author contributions

MK and YR: Had the original idea; MK and YR: Prolonged presence in space and the need for a functional diet; MK, YR, SD: Space farm essentials and constraints; All authors: Ideal candidates for the astronaut's functional salad; All authors: Challenges ahead.

### Conflict of interest statement

The authors declare that the research was conducted in the absence of any commercial or financial relationships that could be construed as a potential conflict of interest.
